# Tannic Acid, A Hydrolysable Tannin, Prevents Transforming Growth Factor-β-Induced Epithelial–Mesenchymal Transition to Counteract Colorectal Tumor Growth

**DOI:** 10.3390/cells11223645

**Published:** 2022-11-17

**Authors:** Mahassen Barboura, Clarisse Cornebise, François Hermetet, Abderrahmane Guerrache, Mouna Selmi, Abir Salek, Leila Chekir-Ghedira, Virginie Aires, Dominique Delmas

**Affiliations:** 1 UFR des Sciences de Santé, Université de Bourgogne, 21000 Dijon, France; 2 INSERM Research Center U1231—Cancer and Adaptive Immune Response Team, Bioactive Molecules and Health Research Group, 21000 Dijon, France; 3 Research Unit Bioactive Natural Products and Biotechnology UR17ES49, Faculty of Dental Medicine of Monastir, University of Monastir, Avicenne street, Monastir 5000, Tunisia; 4 INSERM Research Center U1231—DesCartes Team, 21000 Dijon, France; 5 Centre Anticancéreux Georges François Leclerc Center, 21000 Dijon, France

**Keywords:** polyphenol, tannic acid, colon cancer, EMT, chemoprevention

## Abstract

Despite the medico-surgical progress that has been made in the management of patients with colorectal cancer (CRC), the prognosis at five years remains poor. This resistance of cancer cells partly results from their phenotypic characteristics in connection with the epithelial–mesenchymal transition (EMT). In the present study, we have explored the ability of a polyphenol, tannic acid (TA), to counteract CRC cell proliferation and invasion through an action on the EMT. We highlight that TA decreases human SW480 and SW620 CRC cell and murine CT26 CRC cell viability, and TA inhibits their adhesion in the presence of important factors comprising the extracellular matrix, particularly in the presence of collagen type I and IV, and fibronectin. Moreover, these properties were associated with TA’s ability to disrupt CRC cell migration and invasion, which are induced by transforming growth factor-β (TGF-β), as evidence in the video microscopy experiments showing that TA blocks the TGF-β1-induced migration of SW480 and CT26 cells. At the molecular level, TA promotes a reversal of the epithelial–mesenchymal transition by repressing the mesenchymal markers (i.e., Slug, Snail, ZEB1, and N-cadherin) and re-expressing the epithelial markers (i.e., E-cadherin and β-catenin). These effects could result from a disruption of the non-canonical signaling pathway that is induced by TGF-β1, where TA strongly decreases the phosphorylation of extracellular-signal regulated kinase ERK1/2, P38 and the AKT proteins that are well known to contribute to the EMT, the cell motility, and the acquisition of invasive properties by tumor cells. Very interestingly, a preclinical study of mice with subcutaneous murine tumor colon CT26 cells has shown that TA was able to significantly delay the growth of tumors without hepato- and nephrotoxicities.

## 1. Introduction

Colorectal cancer (CRC) represents a global health threat owing to its high incidence, mortality rates, and morbidity [1]. It is the third most commonly diagnosed cancer and the second most common cause of cancer-related death in both men and women [2,3]. This unfavorable prognosis partly results from the fact that colon cancer is a tumor with a high metastatic propensity. The tumor invasion and metastasis in the middle and late stages are the root causes of treatment failure and poor therapeutic efficacy [4,5]. Among the molecular mechanisms leading to the process of metastasis, the epithelial–mesenchymal transition (EMT) plays a fundamental role. This is a complex dynamic process by which the epithelial cells dedifferentiate into a more mobile mesenchymal cell phenotype. Indeed, the EMT is one of the fundamental molecular steps in the process of tumor progression and distant metastasis, which facilitate the invasion and migration in different cancers, and is correlated with a poor prognosis in CRC [6]. During the EMT, the epithelial cells lose epithelial characteristics such as polarity and specialized cell-to-cell contact, and acquire a mesenchymal phenotype with increased migratory and invasiveness abilities [7]. During this process, the epithelial cells lose the expression of cell–cell adhesion proteins, including E-cadherin and β-catenin, and acquire the expression of mesenchymal markers, including vimentin and N-cadherin [8]. This mechanism is also characterized by the activation of various transcription factors, which include Snail, Slug, and Zinc finger E-box-binding homeobox (ZEB) 1/2 [7]. Snail and Slug are mainly known for their involvement in the EMT, where they repress the expression of the epithelial markers, such as E-cadherin and Claudin-1, and increase the expression of the mesenchymal markers, such as ZEB1 and MMP-9 [9,10]. Several oncogenic pathways that respond to extracellular cues have been shown to contribute to the EMT of the carcinoma cells, such as transforming growth factor-β (TGF-β), bone morphogenetic protein (BMP), Wnt/β-catenin, Notch, and Hedgehog signaling pathways. TGF-β is the first EMT inducer that has been described in normal mammary epithelial cells [11], and it plays a crucial role in tumor metastasis. Thus, the inhibitors of the TGF-β signaling pathway have become attractive tools as strategies to prevent tumor progression [12].

In this context, we and others have shown the potential ability of natural compounds to act in a chemopreventive or a chemosensitization strategy [13,14,15,16,17]. Indeed, many in vitro and in vivo studies in animals have shown that these natural molecules are able to block all of the stages of carcinogenesis and can also sensitize tumor cells to many anticancer agents, thus increasing their chemotherapeutic potential. At the cellular and molecular levels, polyphenols can act at the DNA level and can modulate many of the signaling pathways that are involved in cell death, inflammation, and angiogenesis, as well as in the metabolism [13,18,19,20,21]. Due to their ability to act as anti- and pro-oxidant molecules, polyphenols could also contribute to limiting certain fundamental aspects of tumorigenesis and resistance mechanisms by acting on the cellular redox balance [21]. In later stages, these natural bioactive compounds could also act on multiple cell processes or conditions that are known to be involved in cancer progression, such as altered tumor metabolism and a pro-inflammatory microenvironment, migration, invasion, angiogenesis, and metastatic spread. In relation to this latter aspect, various natural compounds exhibit an inhibitory action on the EMT [22]. Among these natural compounds that could participate to a chemosensitization strategy and act on EMT, tannic acid (TA) could be a good candidate. This hydrolysable tannin is present in several natural sources, such as grapes, green tea, and coffee [23], and a myriad of pharmaceutical and biological applications in the medical field has been well recognized [24]. Among these effects, potential anticancer activities against several solid malignancies, such as lung, liver, pancreatic, breast, ovarian, and colorectal cancers, have been reported [25]. A recent study has shown that, in pulmonary fibrosis, TA could repress TGF-β signaling and the subsequent EMT process in lung epithelial cells [26]. However, the potential effect of TA on the EMT in CRC and its chemopreventive properties still remain to be defined and deserve further investigation.

In the present study, we first investigated whether TA was able to counteract the processes of cell migration and invasion in human SW480 and murine CT26 CRC cells lines. We show the ability of TA to significantly decrease both the adhesion and the TGF-β-induced proliferation of CRC cells. Moreover, TA inhibits the TGF-β1-induced motility of metastatic colon cancer cells in vitro, and this inhibition is associated with a decrease in the mesenchymal biomarkers and an increase in epithelial protein expression. Our in vivo efficacy studies demonstrate a tumor regression in the mice that were supplemented with TA without toxicity. These findings provide new evidence that TA could be a robust and promising tool in the development of innovative chemoprevention strategies.

## 2. Materials and Methods

### 2.1. Cell Culture and Treatments

SW480 (RRID: CVCL_0546), SW620 (RRID: CVCL_0547) human, and CT26 (RRID: CVCL_7254) murine adenocarcinoma cell lines were purchased from ATCC (Rockville, MD, USA) and were maintained in RPMI medium (Life Technologies, Gaithersburg, MD, USA) that was supplemented with 10% fetal bovine serum (FBS) (Life Technologies). All cell lines were cultured in a humidified chamber at 37 °C with 5% CO_2_.

### 2.2. Drugs, Antibodies, and Chemical Reagents

Tannic acid (TA) was obtained from Sigma-Aldrich laboratory (St. Quentin Fallavier, France) and a stock solution was prepared in dimethyl sulfoxide (DMSO). Antibodies targeting E-cadherin, N-cadherin, β-catenin, ZEB1, Slug, Snail, P38 MAPK, phospho (p) P38 MAPK, ERK1/2, phospho (p) ERK1/2, AKT, phospho (p) AKT, and GAPDH were used.

### 2.3. Crystal Violet Staining Assay

Cell viability was determined using crystal violet (CV) (4-[(4-dimethylaminophenyl)-phenyl-methyl]-N,N-dimethyl-aniline) to stain the DNA, as described previously [27,28]. Cells were seeded in triplicates in 96 well plates in RPMI + 10% FBS at the density of 2, 2.5, and 3 × 10^4^, respectively, and incubated for 24 h in 5% CO_2_ atmosphere and 95% humidity at 37 °C. Then, the cells were treated at 24 h, 48 h, and 72 h, with increasing concentrations of TA (1:2 serial dilutions; starting concentration 100 µM). As a control, cells were treated with the vehicle alone (DMSO 0.1%). After the treatment, the cells were washed with phosphate-buffered saline (PBS) 1×, and then fixed and stained with a CV solution (crystal violet 2.3%, ammonium oxalate 0.1%, ethyl alcohol 20%, 5 min), and rinsed with water. After extensive washing of the cells with water, CV was finally solubilised in 33% acetic acid. The absorbance was measured at 590 nm using a BiochromAsys UVM 340 spectrophotometer. The assays were carried out in three independent experiments. The results were expressed as a percentage of cell viability reffering to those treated with vehicle alone (control) assumed as 100% and using the following equation: Cell viability (%) = 100 − [(Absorbance control − Absorbance test/Absorbance control) × 100]. The dose–response curves were then plotted using GraphPad Prism software (v8.3.0), in order to determine, at the different times of treatment, the 50% inhibitory concentrations (IC_50_, concentration of a molecule inducing 50% of toxicity on a given cell population) after an adjustment of the data by a non-linear regression with 4 parameters.

### 2.4. Cell Adhesion Assay

Cell adhesion was determined using a method described in [29]. Briefly, 96-well plates were pre-coated with fibronectin (1 μg/mL, Sigma-Aldrich, St. Louis, MO, USA) in Hank’s buffer (Welgene Inc., Daegu, Republic of Korea), collagen I (20 µL, 5 g/mL, 0.01 M HCl in PBS), collagen IV (10 μg/mL in PBS), or poly-L-lysine (50 μg/mL), and allowed to adhere for 1 h at 37 °C. BSA (1% diluted in PBS) was added to each well for 1 h. The wells were washed twice with PBS and left to dry for a further 2 h. The cells were suspended in serum complete media containing TA at different concentrations and then plated into wells. After 48 h, the non-adherent cells were removed by media aspiration. The adherent cells were stained with 0.1% CV for 10 min at room temperature (RT), dissolved with 1% sodium dodecyl sulfate (Sigma-Aldrich), and then quantified by measuring the absorbance at 540 nm using a plate reader (Bio-Rad Laboratories).

### 2.5. Migration Using Wound-Healing Assay and Incucyte Device

A wound-healing assay was carried out to evaluate the effect of TA on cell migration. Cells were grown in six-well plates overnight until confluence. When the cells reached confluency, a wound was made by scratching the monolayer of cells using a sterile plastic 10 µL pipette tip. Then, the cells were rinsed with PBS to remove any free-floating cells and debris. Serum-free cell medium was then added, and culture plates were incubated at 37 °C in the presence of TA for 48 h. Images of the scratched areas were captured with an inverted microscope (Olympus IX-71, Manchester, UK). Experiments were performed in triplicate for each condition and cell line. The average widths of the wounds at 0 h, 24 h, and 48 h time points were measured using Image-J software (NIH). The distance (D) migrated by the cells was calculated as follows: D = (size of the wound at t = 0 h—size of the wound at t = 24 h or 48 h). We next evaluated the effect of TA on TGF-β1-induced cell migration using the *IncuCyte*^®^ *S3* Live-Cell Analysis System (Essen BioScience, Ann Arbor, MI, USA). The SW480 and CT26 cells were seeded in 96-well plates at 8 × 10^4^ and 3 × 10^5^ cells/well, respectively, in 100 µL of complete RPMI and incubated for 6 h at 37 °C and 5% CO_2_. Then, they were serum starved (0.5% FBS) overnight. A homogeneous wide wound in the cell monolayer of each well was performed using the Wound Maker (Essen BioScience, Ann Arbor, MI, USA). The cells were washed twice with HBSS and then treated with TGF-β1 (10 ng/mL) in the presence or absence of TA (0.8 µM) for 72 h. The untreated cells served as the controls (solvents alone, Co). The plate was placed at 37 °C in the presence of 5% CO_2_ in the *IncuCyte*^®^system and the cell migration was recorded every 2 h by phase-contrast scanning (10× objective) for 72 h. The wound recovery area at each time point was analyzed and the percentages of the wound relative density was determined with the using Incucyte^®^*S3* Software update (V2021B).

### 2.6. Migration and Invasion Using Transwell Assay

Cells were seeded in Transwell^®^ chambers with an 8.0 µm Pore Polyester Membrane Insert (3464, Corning, Fisher Scientific S.A.S, Illkirch, France) for migration and invasion assays. For the migration assay, the treated cells in the serum-free medium were plated in uncoated inserts and incubated for 48 h. For the invasion assay, the inserts were pre-coated with 100 μL of Matrigel^®^ (Matrigel^®^ Basement Membrane Matrix, 354,234, Corning, NY, USA), and the treated cells were plated in the serum-free medium, as described above, for an incubation period of 48 h. A total of 500 µL of culture medium containing 10% FBS was added to the lower chamber. The cells attached to the bottom of the membrane were fixed with 4% paraformaldehyde, then were stained with 5% CV (Sigma-Aldrich). After 2 washes of the membrane, the CV was finally solubilized in 33% acetic acid. The absorbance was measured at 590 nm using a BiochromAsys UVM 340 spectrophotometer. Three independent experiments were performed in triplicates.

### 2.7. Western Blotting

Western blotting analysis was performed as described previously [30]. Cells were seeded into 75 cm^2^ flasks 24 h before treatment, then were serum starved (1% FBS) overnight. The following day, the SW480 and the CT26 cells were treated with solvents alone (control cells) or were co-treated with TA (0.8 µM) and/or TGFβ-1 (10 ng/mL) for 48 h. After treatment, the cells were lysed with radio-immunoprecipitation assay (RIPA) buffer supplemented with a complete phosphatase and protease inhibitor cocktail (Roche, Boulogne Billancourt, France). An equal amount of protein was resolved by SDS-PAGE and transferred to nitrocellulose membranes (Amersham, Les Ulis, France). The membranes were saturated with 5% (*w/v*) nonfat dry milk for 1 h, and incubated with primary antibodies against N-cadherin (1:1000), E-cadherin (1:1000), β-catenin (1:1000), Slug (1:1000), Snail (1:1000), ZEB1 (1:1000), ERK1/2 (1:1000), p-ERK1/2 (1:1000), AKT (1:1000), p-AKT (1:1000), P38 (1:1000), p-P38 (1:1000), and GAPDH (1:3000) overnight at 4 °C. Primary antibodies were detected using horseradish peroxidase (HRP)-conjugated appropriate secondary antibodies (Cell Signaling Technologies, Ozyme, Saint-Cyr-l’Ecole, France), followed by exposure to Enhanced Chemiluminescence (ECL) (Santa Cruz Biotechnology, Heidelberg, Germany). A signal was acquired with a ChemiDocTM XRS + imaging system (Bio-Rad, Marnes-la-Coquette, France), and blots were analyzed with Image Lab^TM^ v6.0.1 software (Bio-Rad, Marnes-la-Coquette, France).

### 2.8. Animal Studies

A total of 15 male BALB/c mice (8 weeks old and weighing ≈ 25 g) were housed according to the Council of the European Communities (86/609/EEC; 24 November 1986) Directives regulating the welfare of experimental animals, and experiments were approved by the Life Sciences and Health Research Ethics Committee (cer-svs) of the Institute of Biotechnology (University of Monastir, Tunisia; ethical approval no. 2021/02/I/CER-SVS/ISBM; 9 January 2021). The mice were maintained in a pathogen-free environment (24 °C and 50% humidity) on a 12 h light/12 h dark cycle, with food and water supplied ad libitum throughout the experimental period. The mice were allowed to acclimatize under the laboratory conditions for 1 week before being used for the experiments. CT26 colon cancer cells (10^6^, suspended in 200 μL of PBS) were subcutaneously (s.c.) injected into their right hind leg. One week after tumor cell injection, the mice were separated into the following three experimental groups: vehicle (as a control), TA, and 5-FU (n = 5 per group). For all groups, DMSO was used at the final concentration of 0.1% and the control group received a saline solution with 0.1% of DMSO (Sigma-Aldrich, St. Quentin Fallavier, France, reference 67-68-5). In group 1, mice were treated with TA diluted in 0.1% DMSO, which was administered 3 times per week by intraperitoneal injection (15 mg/kg). In group 2, mice were treated with 5-FU in 0.1% DMSO once per week by intraperitoneal injection (15 mg/kg). The mice in the control group were administered a sterile saline solution with the same timing and dosing schedule as that used for the other treatment groups. The tumor volumes (TV) and body weights of the mice were measured every 2 days. TV was calculated using the following formula: TV (mm^3^) = ½ × (D × d^2^), where D is the longest and d is shortest diameter.

### 2.9. Biochemical Assay

Blood samples were collected from the sacrificed mice. The serum samples were analyzed by the Biochemistry Department laboratory of the University Hospital Center (UHC) FarhatHached (Sousse, Tunisia). The aspartate transaminase (ASAT), alanine transaminase (ALAT), and creatinine (CR) levels were determined by automated analysis using a commercial Cobas Integra kit (Roche, Boulogne-Billancourt, France).

### 2.10. Satistical Analyses

The data are expressed as mean ± standard deviation (SD), or standard error of the mean (SEM), as indicated in the figure legends. Statistical analysis was carried out with Prism GraphPad8.0 Prism software (GraphPad Software, La Jolla, San Diego, CA, USA). Statistical comparisons among groups were performed using one-way and two-way analysis of variance (ANOVA), followed by Tukey’s test for multiple comparison. The *p*-values ≤ 0.05 were considered significant (* *p* < 0.05, ** *p* < 0.01, *** *p* < 0.001, and **** *p* < 0.0001).

## 3. Results

### 3.1. TA Inhibits the Proliferation of Colorectal Carcinoma Cells

**Figure 1:**
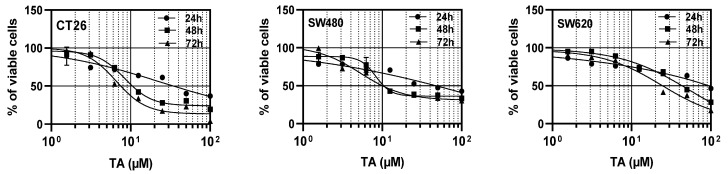
TA decreases the viability of CT26, SW480, and SW620 CRC cells in a dose- and time-dependent manner. Cells were exposed to increasing concentrations of TA (from 0 to 100 µM) for 24 h, 48 h, and 72 h. Cell viability was determined by CV assay and expressed as a percentage of the control (DMSO). Dose–response curves were plotted at the different treatment time points in the 3 cell lines with 4-parameter non-linear regression using GraphPad prism software. Each point of the curves represents the mean percentage of viable cells ± SD of 3 independent experiments, with 5 replicates per condition.

In order to determine whether TA presents potential chemopreventive properties against CRC, we first evaluated its cytotoxic effect on two human colorectal carcinoma cells lines, which were SW480 and its metastatic derived cell line SW620, and a murine CT26 CRC cell line was used in order to further explore the effect of TA in an in vivo model. Using the CV assays, we observed that TA induced a dose- and time-dependent cytotoxic effect, the latter of which being more pronounced from 48 h of treatment, using TA concentrations ranging from 0 to 100 µM (Figure 1). Indeed, the inhibitory concentration of 50% (IC_50_) that was obtained after 48 h of treatment was found to be of 12 µM and 44 µM for SW480 and SW620, respectively, and was of 11 µM for the murine CT26 cells*,* the latter of which being the most sensitive cell line (Figure 1, Table 1). Based on these values at 48 h of treatment, we chose the following IC_50_-related doses for subsequent experiments: 0.5 fold IC_50_ (1/2 IC_50_), IC_50_, and 2 fold IC_50_ (2 IC_50_). In order to study the EMT, we focused on the two cell lines that were found to be most sensitive to TA, which were the SW480 and CT26 cells. The SW620 cells displayed lower sensitivity to TA, and as they have a metastatic phenotype (they are derived from a metastasis of the same tumor from which SW480 cells are derived), they were not used in the following experiments aiming to evaluate the inhibitory potential of TA on the EMT.

### 3.2. TA Inhibits Colorectal Carcinoma Cell Adhesion

**Figure 2:**
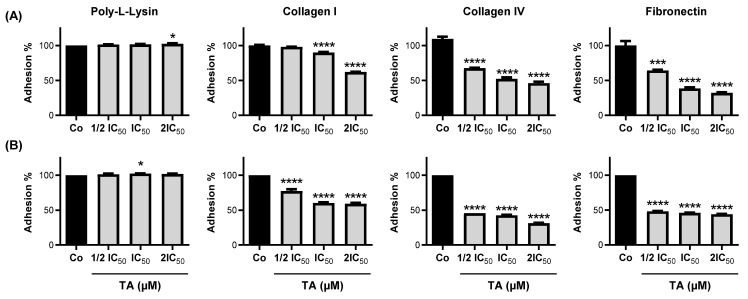
TA inhibits CRC cell adhesion. SW480 (**A**) and CT26 (**B**) cells were exposed for 30 min to 3 different IC_50_-related doses of TA (SW480 cells: IC_50_ = 12 µM, ½ IC_50_ = 6 µM, and 2IC_50_ = 24 µM; CT26 cells: IC_50_ = 11 µM, ½ IC_50_ = 5.5 µM, and 2IC_50_ = 22 µM) and seeded on poly-L-Lysin, type I or type IV collagen (collagen I and collagen IV, respectively), and fibronectin pre-coated wells to evaluate ECM protein-dependent cell adhesion. After 48 h, the percentage of attached cells was determined by CV assay. The results are expressed as the percentage of solvent control (DMSO, Co) (mean ± SD) of 3 independent experiments performed in triplicate per condition. Statistical significance was determined by one-way ANOVA, followed by Tukey’s test for multiple comparison tests, with *p* < 0.05 (*), *p* < 0.001 (***), and *p* < 0.0001 (****) vs. the corresponding control group.

Before studying the EMT, we evaluated the capacity of TA to modulate the adhesion of CRC cells in the presence of three important factors of the extracellular matrix (ECM). Indeed, for example, an ECM that is rich in type I collagen has been shown to confer a tumor phenotype to untransformed pancreatic ductal cells [31]. In a same manner, fibronectin induces the EMT in CRC cells and plays a pivotal role in promoting CRC metastasis [32]. A recent review has summarized the ECM content in the stroma surrounding the primary CRC and presented a quantitative comparison of the identified components with those from a normal colon [33]. In the light of these elements, we tested the efficiency of TA to decrease the adhesion capacity of CRC cells in the presence of the following three major compounds, that are found increased during EMT process: collagen type I and IV, and fibronectin. We next evaluated the specific adhesion and the nonspecific cell adhesion using collagen I and IV or fibronectin pre-coated wells, respectively, and poly-L-lysine pre-coated wells. The CRC cells’ capacity to adhere to these differentially pre-coated wells was evaluated after 48 h of seeding and with a prior treatment of the cells for 30 min in the presence or absence of TA, which was used at three concentrations, based on the IC_50_ values that were determined at 48 h (Table 1). In these experimental settings, we observed that TA was able to significantly decrease the percentage of cell adhesion only in the presence of the collagen I and IV, and fibronectin for the SW480 and CT26 cell lines in a concentration-dependent manner (Figure 2). It is interesting to note that no effect was observed on cell adhesion to the integrin-independent substrate, the poly-L-lysine.

### 3.3. TA Inhibits Cell Migration

**Figure 3:**
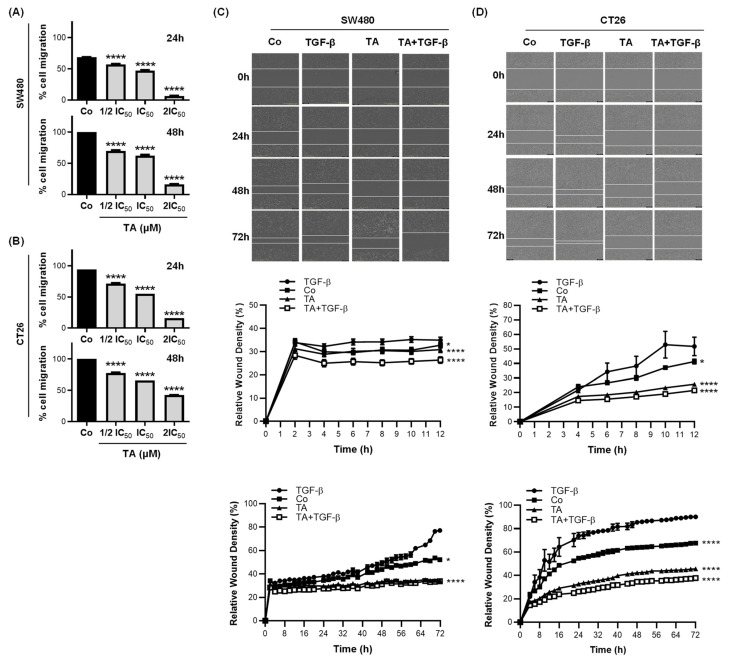
TA decreases the basal and TGF-β-induced migratory abilities of CRC cells. (**A**,**B**) Confluent SW480 and CT26 cell monolayers were subjected to scratch wounding and then cultured in RPMI medium with 0.5% FBS only (Control, Co), or in the presence of TA (SW480 cells: IC_50_ = 12 µM, ½ IC_50_ = 6 µM, and 2IC_50_ = 24 µM; CT26 cells: IC_50_ = 11 µM, ½ IC_50_ = 5.5 µM, and 2IC_50_ = 22 µM). The percentages of the wound recovery area at 24 h and 48 h, reflecting cell migration, were calculated as described in the Materials and Methods Section and are expressed as the mean ± SD of 3 experiments (n = 3) carried out in 3 replicates per condition. Statistical significance was determined by one-way ANOVA, followed by Tukey’s test for multiple comparison tests, with **** *p* ≤ 0.0001, vs. the corresponding control group. (**C**,**D**) Confluent SW480 and CT26 cell monolayers were subjected to scratch wounding and then cultured in RPMI medium with 0.5% FBS only (Control, Co), or in the presence of TA (0.8 µM), TGF-β (10 ng/mL), or TA and TGF-β (TA + TGF-β). The percentages of the wound recovery area over time (up to 72 h), reflecting cell migration, were calculated as described in the Materials and Methods Section and are expressed as the mean ± SD of 3 experiments (n = 3) carried out in 3 replicates per condition. (**C**,**D**, top panel). Representative photomicrographs (10× objective) of the wounds at 0 h (initial wounds), and at 24 h, 48 h, and 72 h were taken using the IncuCyte^®^ S3 Live-Cell Analysis System. The dotted lines represent the migration front lines. Statistical significance was determined by one-way ANOVA, followed by Tukey’s test for multiple comparison tests, with * *p* < 0.05, **** *p* < 0.0001, vs. TGF-β group.

**Figure 4:**
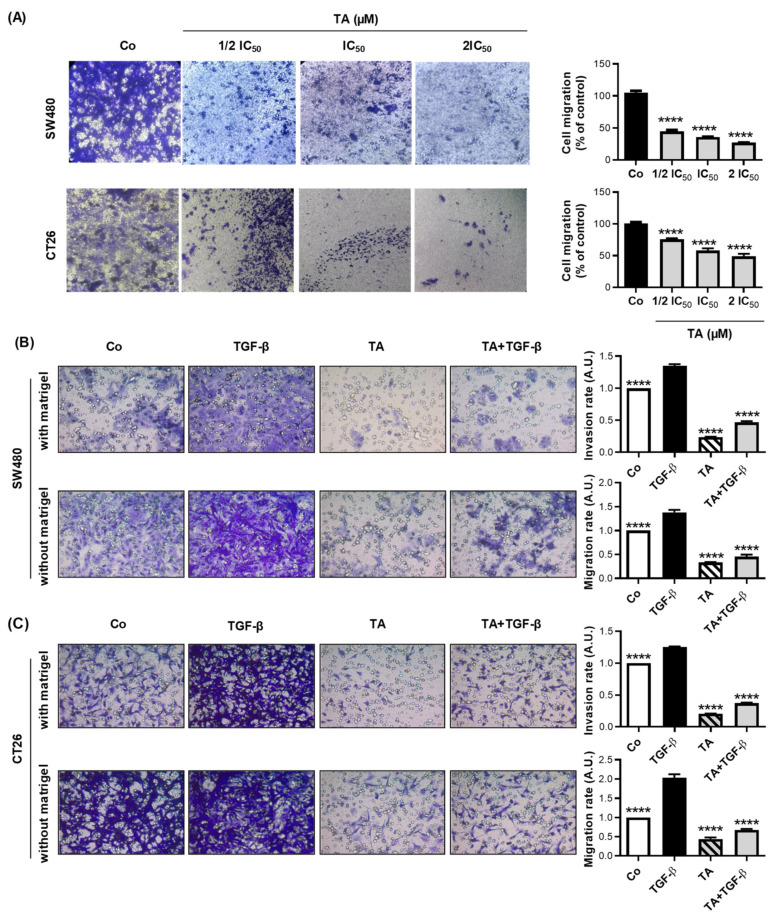
TA decreases the basal and TGF-β-induced migration abilities and invasiveness of CRC cells. SW480 and CT26 cells were seeded into the upper filters (migration assay) or on Matrigel-precoated filters (invasion assay) of Boyden chambers and incubated at 37 °C in complete RPMI medium only (Control, Co), or in the presence of TA (0.8 µM), TGF-β (10 ng/mL), or a combination of both (TA + TGF-β). After 48 h, cells that had migrated to the bottom of the filter were stained using CV and photographed. The cell migration ability was determined by measurement of CV absorbance at 540 nm after solubilization in 33% acetic acid. (**A**) Representative photomicrographs (10× objective) of the trans-well migration assays on the left panel. The right panel represents the cell migration rate normalized as a percentage of control (100%). Representative photomicrographs (10× objective) of the trans-well Matrigel invasion (upper row) and migration (bottom row) assays are on the left panel for (**B**) SW480 and (**C**) CT26 cell lines. The right panel represents the cell invasion (upper) and migration (bottom) rates normalized to the control. Results are expressed as mean ± SD of 3 independent experiments (n = 3) carried out in 3 replicates per condition. Statistical significance was determined by one-way ANOVA, followed by Tukey’s test for multiple comparison tests, with **** *p* ≤ 0.0001, vs. the corresponding control group.

Metastasis development involves multiple biological mechanisms, including an increase in cell motility. For this reason, the migratory capacity of SW480 and CT26 cells was assessed with wound-healing assays. Confluent cell monolayers were scratched and then the recolonization of the latter was monitored at specific time points (24 h and 48 h) by microscopy or by video microscopy for 72 h. The concentrations of TA that were used for the wound-healing treatments were ½ IC_50_, IC_50_, and 2IC_50_, as determined previously in Figure 1 and Table 1 for the two cell lines. As shown in Figure 3, TA inhibited SW480 and CT26-cell migration after 48 h of incubation in a dose-dependent manner, with an inhibition rate of >80% in SW480 and 50% in CT26. In addition, the result from the trans-well migration assay was in line with the data from the scratch assay. In Figure 4A, TA efficiently inhibited SW480 and CT26-cell migration in a dose-dependent-manner and the inhibition rates after 48 h of treatment were >72% in SW480 and 50% in CT26 cells.

### 3.4. TA Inhibits TGF-β1-Induced Motility of Metastatic Colon Cancer Cells In Vitro

The effects of TA on TGF-β1 induced cell migration were further studied using a real-time cell imaging system (IncuCyte^®^ S3 Live-Cell Imaging System, Essen BioScience, Ann Arbor, MI, USA). As shown in Figure 3C,D, TA blocked the TGF-β1-induced migration of the SW480 and CT26 cells. Through the EMT, the cells acquire migratory and invasive capacity. The migration and invasion of cells are the essential steps to form metastatic foci. Thus, we next determined the effects of TA on the invasion and migration that was induced by TGF-β1. As shown in Figure 4B,C, TA blocked the TGF-β1-induced invasion and migration of the SW480 and CT26 CRC cells.

### 3.5. TA Restores Epithelial Markers Expressionin CRC Cells

**Figure 5:**
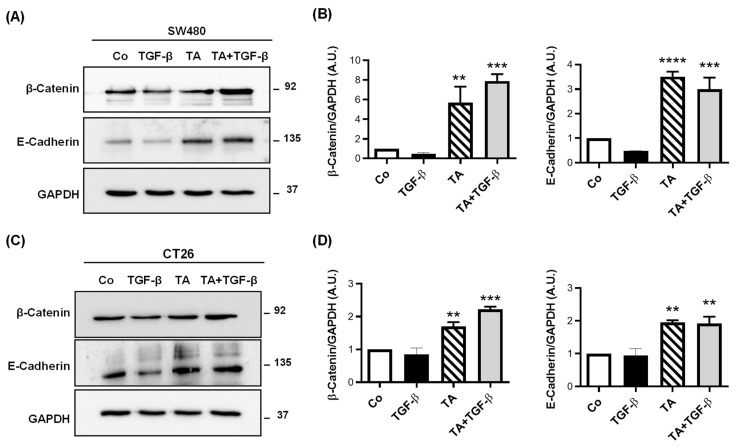
TA inhibits the TGF-β-induced decrease in epithelial marker expression in SW480 and CT26 cells. Serum-starved SW480 and CT26 cells were treated with TA (0.8 µM), TGF-β (10 ng/mL), or a combination of both (TA + TGF-β) for 48 h. Vehicle-treated cells served as the control (Co). Representative immunoblot and densitometry quantification analysis of EMT markers β-catenin and E-cadherin in SW480 (**A**,**B**) and CT26 (**C**,**D**) after 48 h of treatments. The band intensity was quantified using GAPDH as an internal loading control, and quantification values were normalized to the control. Results are expressed as mean ± SD of 3 independent experiments (n = 3). Statistical significance was determined by one-way ANOVA, followed by Tukey’s test for multiple comparison tests, with ** *p* < 0.01, *** *p* <0.001, and **** *p* < 0.0001, vs. the corresponding TGF-β1 group.

The migratory capacity of cancer cells is necessary for metastasis and the acquisition of metastatic capability is associated with the EMT in cancer cells. During the process of EMT, the epithelial cells lose cell-to-cell contacts and gain the expression of mesenchymal factors, enabling migration and invasion into the surrounding stroma in order to facilitate metastasis. In this process, E-cadherin and β-catenin loss are relatively common in cancers of epithelial origin, such as colon cancer. In order to investigate the effect of TA on the TGF-β1-induced EMT, the protein expression of the epithelial markers, such as β-catenin and E-cadherin, was analyzed in SW480 and CT26 cells (Figure 5).

First of all, we observed that TA, at the IC_50_, significantly increases the protein expression of β-catenin and E-cadherin after 48 h of treatment in SW480 cells (Figure 5A,B). Very interestingly, when SW480 cells are cotreated with TGF-β1 and TA over 48 h, the increases in the two epithelial markers are maintained at the induction levels similar to those that were obtained with TA alone (Figure 5A,B). In the same way, similar results are observed in CT26 cells, with a weak but significant TA of β-catenin and E-cadherin expression, compared to the control (Figure 5C,D). As observed in the SW480 cells, the protein expression level of β-catenin and E-cadherin was very similar to TA alone when cotreated with TA and TGF-β1 in the CT26 cells (Figure 5C,D).

### 3.6. TA Inhibits Mesenchymal Marker Expression in Colorectal Cancer Cells

**Figure 6:**
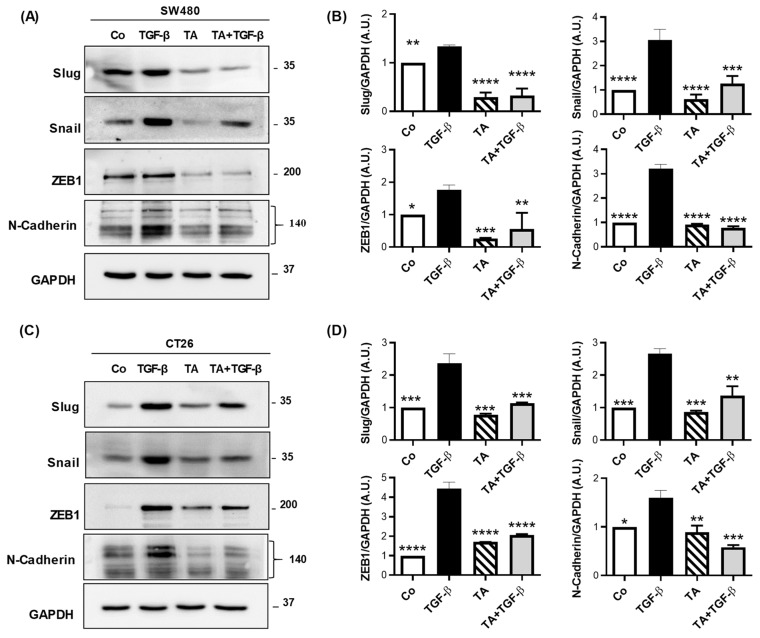
TA inhibits TGF-β-induced mesenchymal marker expression in SW480 and CT26 cells. Serum-starved SW480 and CT26 cells were treated with TA (0.8 µM), TGF-β (10 ng/mL), or a combination of both (TA + TGF-β) for 48 h. Vehicle-treated cells served as the control (Co). Representative immunoblot and densitometry quantification analysis of EMT markers Slug, Snail, ZEB1, and N-cadherin in SW480 (**A**,**B**) and CT26 (**C**,**D**) after 48 h of treatment. The band intensity was quantified using GAPDH as an internal loading control, and quantification values were normalized to the control. Results are expressed as mean ± SD of 3 independent experiments (n = 3). Statistical significance was determined by one-way ANOVA, followed by Tukey’s test for multiple comparison tests, with * *p* ≤ 0.05, ** *p* < 0.01, *** *p* < 0.001, and **** *p* < 0.0001, vs. the corresponding TGF-β1 group.

Mesenchymal markers, such as Slug, Snail1, and ZEB1, enable EMT through various mechanisms [34]. They act by either repressing the transcription of genes coding for proteins of adherent junctions, such as E-cadherin [35], or by activating the expression of mesenchymal genes (N-cadherin, vimentin), which are involved in cytoskeleton remodeling and enzyme production, promoting cell motility and basal lamina degradation. In the present study, we highlight that TA was able to both decrease the protein expression of the transcription factors Slug, Snail1, and ZEB1 in basal conditions and also counteract their TGF-β1-induced expression (Figure 6).

The transcription factors, Slug, Snail, and ZEB1 were significantly decreased with TA treatment alone, as compared to the control by 70, 40, and 65%, respectively, in SW480 cells (Figure 6A,B). As expected, and previously described in the literature, TGF-β1 induces a strong expression of all of the transcriptional factors (Slug, Snail, and ZEB1), as well as N-cadherin (Figure 6A,B). Interestingly, when SW480 cells are cotreated with TGF-β1 and TA over 48 h, we observed a strong inhibition of all of the TGF-β1-induced epithelial markers in the SW480 cells by 90% (Slug), 60% (Snail), 68% (ZEB1), and 78% (N-cadherin), as compared to TGF-β1 alone (Figure 6A,B). These effects of TA on TGF-β1-induced epithelial markers in the SW480 cells were also observed in the CT26 cells (Figure 6C,D). Overall, our data provide in vitro evidence that TA prevents the epithelial cells from transitioning to a mesenchymal-like phenotype induced by TGF-β1 treatment.

### 3.7. TA Antagonizes TGF-β1-Mediated Smad-Independent Signaling Pathways

Activation of Smad-dependent and/or independent signaling pathways are reported to be critical for TGF-β-induced EMT [36]. In order to further explore the mechanism underlying the antifibrotic effects of TA, overnight serum-starved SW480 and CT26 cells were stimulated with TGF-β1 for 48 h in the presence or absence of TA. We investigated the influence of TA on other non-Smad pathways, such as mitogen-activated protein kinase (MAPK), signaling (P38 and ERK), and AKT signaling pathways. 

**Figure 7:**
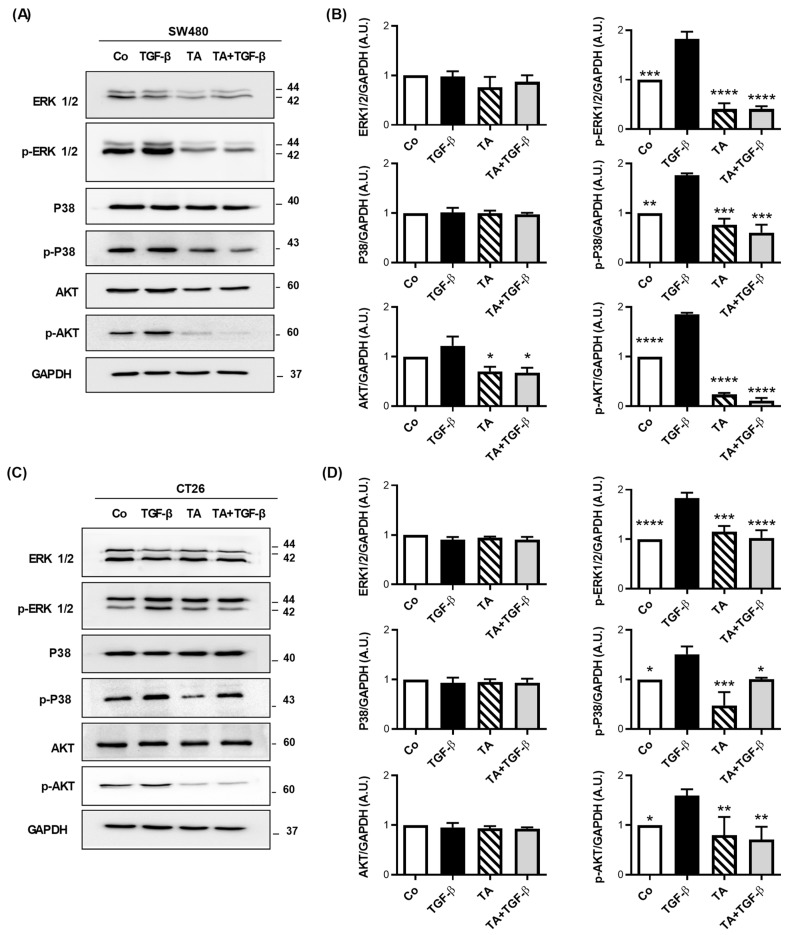
TA blocks TGF-β-induced non-Smad-dependent signaling pathways in SW480 and CT26 cells. Serum-starved SW480 and CT26 cells were treated with TA (0.8 µM), TGF-β (10 ng/mL), or a combination of both (TA + TGF-β) for 48 h. Vehicle-treated cells served as the control (Co). Representative immunoblot and densitometry quantification analysis of total and phosphorylated (*p*-) ERK_1/2_, P38, and AKT in SW480 (**A**,**B**) and CT26 (**C**,**D**) cells after 48 h of treatments. The band intensity was quantified using GAPDH as an internal loading control, and quantification values were normalized to the control. Results are expressed as mean ± SD of 3 independent experiments (n = 3). Statistical significance was determined by one-way ANOVA, followed by Tukey’s test for multiple comparison tests, with * *p* < 0.05, ** *p* < 0.01, *** *p* < 0.001, and **** *p* < 0.0001, vs. the corresponding TGF-β1 group.

Firstly, as expected, the TGF-β1 treatment alone induced the phosphorylation of ERK1/2, P38, and AKT protein, without modulation of their expression level (Figure 7). Very interestingly, the treatment of SW480 and CT26 cells for 48 h with TA alone, or in combination with TGF-β1, strongly decreased phosphorylated forms of ERK1/2, P38, and AKT (Figure 7). Collectively, our results have indicated that TA inhibits TGF-β1-induced EMT through the inhibition of non-Smad signaling pathways.

### 3.8. TA Prevents Murine Colon Carcinoma Growth 

**Figure 8:**
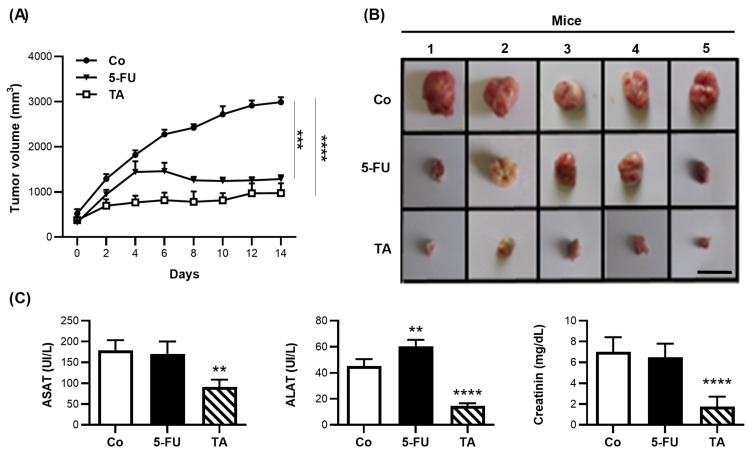
TA inhibits tumor growth without hepato- and nephrotoxicities in vivo. (**A**) One week after tumor cell injection, CT26 subcutaneous tumor-bearing BALB/c mice were treated (day 0 on the graph) with sterile saline solution (control group (Co, 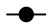), 5-FU (15 mg/kg, 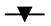), or TA (15 mg/kg, 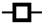), (n = 5 mice per group)), and the treatment was continuing at a rate of one injection every 2 days. The evolution of tumor volumes (mm^3^) in time was determined. (**B**) Images of tumors harvested from the mice on day 14 (scale bar = 1 cm). (**C**) Measurement of serum aspartate transaminase (ASAT), alanine transaminase (ALT), and creatinine levels in Co, 5-FU-, or TA-treated mice. The data are (**A**) medians ± SEM or (**C**) means ± SD of 3 independent experiments (n = 3). Statistical significance was determined by one-way ANOVA, followed by Tukey’s test for multiple comparison tests, with ** *p* < 0.01, *** *p* < 0.001, and **** *p* < 0.0001, vs. the corresponding control group.

In order to determine whether TA is able to prevent tumor progression, we used a syngeneic model of CT26 tumors that were subcutaneously injected into BALB/c, which we challenged three times per week, either with a fixed amount of TA (15 mg/kg), or 5-FU (15 mg/kg) or with sterile saline solution for the control group. Our results showed that, comparatively to the control mouse group, TA was able to significantly delay the growth of tumors (Figure 8A,B). As expected, the 5-FU-related side effects were associated with rises in alanine transaminase (ALT), aspartate transaminase (ASAT), and creatinine (CR) levels in the blood samples from mouse group that was treated with the chemotherapeutic agent (Figure 8C). Very surprisingly, the treatment with TA decreased the expression of these three markers of toxicity (Figure 8C), suggesting that the antitumor effect of TA was not associated with a toxicity, as observed in the 5-FU-treated mice (Figure 8C).

## 4. Discussion

Colorectal cancer (CRC) is the second most lethal cancer and the third most prevalent malignant tumor worldwide [3]. Despite the innovation and supportive care in cancer over the last several decades, which increase the overall survival rate of patients due to better cancer prevention, early detection, and treatments, the prognosis of CRC remains unsatisfactory, especially for patients with metastatic lesions [37].

This therapeutic failure can result from numerous phenomena that are linked to the host, to the cancerous cells themselves, which can present an intrinsic or an acquired resistance, or even to the tumor microenvironment. Many studies demonstrate that this would play a primordial role in the progression of the metastatic phenotype and in the acquisition of the phenomenon of chemoresistance.

The re-emergence of nutraceutical and plant/diet extracts, or the formulation of a mixture of them, as a promising field of investigation for the identification of innovative anticancer treatments provide a challenging opportunity to further explore the link between nutritional agents and tumor growth and spreading metastasis.

**Figure 9:**
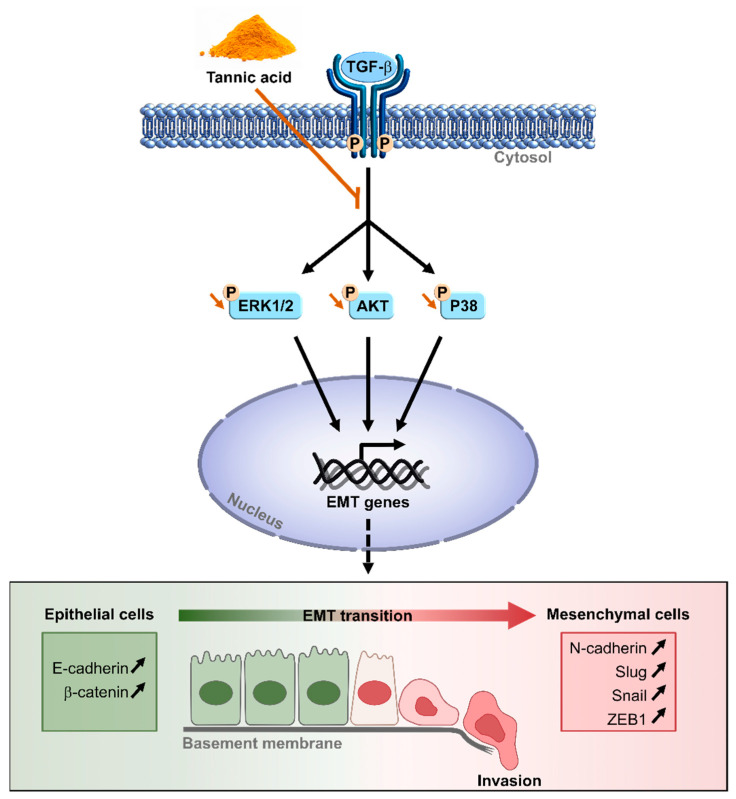
Tannic acid inhibits the epithelial–mesenchymal transition (EMT) of CRC cells by disrupting TGF-β1-mediated Smad-independent signaling pathways. Briefly, tannic acid decreases the phosphorylation of ERK1/2, AKT, and P38, and subsequently prevents invasion mediated by EMT.

In this context, we show for the first time that a polyphenol, such as TA, could affect the microenvironment through a disruption of the EMT in CRC cells. We provide a mechanism by which TA affects the migration and the adhesion of CRC cells via a decrease in mesenchymal biomarkers and an increase in epithelial proteins under TGF-β1 treatment. These events were associated with a TA-induced inhibition of the key regulators of the non-Smad pathways, such as mitogen-activated protein kinase (MAPK), signaling (P38 and ERK), and AKT signaling pathways (Figure 9). By these methods, the supplementation of mice with TA led to tumor regression without toxicity.

EMT is characterized by the occurrence of morphological and phenotypic changes allowing cancer cells to acquire the properties of mesenchymal cells. Recent work suggests the involvement of the EMT in the phenomenon of chemoresistance in CRC [38,39]. From a molecular point of view, the establishment of the mesenchymal phenotype requires modifications of the genetic program that is carried out by ZEB1, Twist, and Snail [40]. In the epithelial cells, the transcription factor Snail is phosphorylated on serine residues by glycogen synthase kinase 3 beta (GSK3β), which constitutes a poly-ubiquitination signal, leading to the degradation of Snail by the proteasome [41]. In the present study, we show that TA was able to strongly decrease both the basal expression of Snail and ZEB1, compared to untreated SW480 and CT26 CRC cells, as well as their expression that is induced by the TGF-β1 (Figure 6). Snail regulates the expression of a large number of proteins. It decreases the expression of the epithelial markers, such as E-cadherin, Claudin-1, and occludin, and activates the expression of the mesenchymal markers, such as fibronectin, vitronectin, and N-cadherin [42]. The transcription factor ZEB acts as a repressor of the transcription of genes coding for the cell junction proteins [43] and also stimulates the expression of N-cadherin [42]. Thus, by its action on Snail and ZEB1, TA also decreases the basal expression of N-cadherin and the expression that is induced by TGF-β1 (Figure 6). Very interestingly, TA affects another important mesenchymal factor, the Slug protein, which has been found to be overexpressed in many cancers, including breast and lung cancers, leukemia, glioblastoma, and hepatocarcinoma, but also CRC [22,24,26,44,45]. This protein contributes to the loss of cell adhesion and polarity by repressing the adhesion proteins. These alterations give the cells the ability to migrate and invade the surrounding tissues and neighboring organs. One of its transcriptional targets is E-cadherin [42]. It is well known that Slug represses the expression of various epithelial genes, such as cytokeratins, occludin, and zonulaoccludens, which are genes that are involved in tight junctions. This transcription factor also initiates the EMT by altering the organization of the desmosomes by repressing the expression of desmoplakin and desmoglein, as well as the cadherins, which are the constituent elements of the desmosomes [46]. In addition, it can activate the transcription of the mesenchymal genes indirectly or directly, such as fibronectin, vimentin, and N-cadherin, depending on the cell type and the cell signals that are transduced [42]. Some studies have demonstrated the existence of cooperation between EMT transcription factors in the inhibition of E-cadherin. Indeed, one of the transcriptional targets of Slug is the ZEB1 protein, which is capable of directly activating the transcription of N-cadherin and vimentin in melanoma [47]. Likewise, TA impacts N-cadherin by decreasing its protein expression and, conversely, by increasing that of epithelial E-cadherin, both at a basal level and under TGF-β1 stimulation (Figure 5 and Figure 6). The E-cadherin is usually located in the adherent junctions and in the basolateral plasma membrane, where, by its extracellular end, it allows interactions between two neighboring cells. Within the same time, the intracytoplasmic region of E-cadherin interacts with the actin cytoskeleton via α- and β-catenins [48]. This complex, therefore, ensures cellular stability and enables the reorganization of the cytoskeleton. In addition to its intercellular adhesion function, E-cadherin transmits signals by interacting with β-catenin [49]. The repression of E-cadherin transcription is mediated by transcription factors, including Snail1, Snail2, ZEB1, ZEB2, and Twist. These E-cadherin transcriptional repressors are generally expressed after the activation of the signaling pathways that are mediated by EMT-inducing factors (i.e., TGF-β1). Consequently, TA increased both E-cadherin and β-catenin in the CRC cells that were used in this study (Figure 5).

Moreover, TA action on Slug is likely to be linked to its anti-migration and anti-invasion properties. Indeed, Slug has been associated with a wide spectrum of biological functions, including motility, migration, invasion, adipogenesis, stem phenotype, and survival. In pathophysiological conditions, these mechanisms contribute to promoting tumor progression, aggressiveness, and also the emergence of metastases. The EMT aids tumor cells in their transition from an epithelial morphology to a motile and invasive mesenchymal phenotype, and then assists tumor cells to cross or disrupt the basal lamina barrier and invade the neighboring tissue. Several studies showed the anti-migratory and anti-invasiveness potential of TA in breast, prostate, and non-small-cell lung cancer cells [44,45,50,51,52]. To the best of our knowledge, our study is the first one that demonstrates that TA significantly abrogated the TGF-β1-induced migration and the invasion capability of CRC cells in vitro, using both murine CT26 and human SW480 cell lines.

Among the signaling pathways that are involved in the EMT process, many are those activated by the TGF-β receptor (TGF-βR). This can stimulate the PI3K/AKT and Smad2/3 pathways that are involved in the repression or the activation of genes coding for proteins involved in the EMT [7]. The TGF-βR signaling pathways lead to the dissolution of cell junctions and, subsequently, to cellular morphological changes [53]. TGF-β1 can also induce the MAPK pathway that is involved in the EMT [54]. In this context, TGF-β1 triggers the activation of the MAPKs, ERK1, and ERK2, in order to promote the expression of Snail and Rho-GTPases, which are a family of G proteins that are involved in the dynamics of the actin cytoskeleton during the migration process. Thus, the MAPK pathway contributes to the establishment of the EMT, cell motility, and the acquisition of the invasive properties of tumor cells [55]. Similar activation of the PI3K/AKT pathway leads to the inhibition of the GSK3β protein, which subsequently favors the stabilization of β-catenin and the transcription factor, leading ultimately to the modulation of the genes controlling the EMT. Very interestingly, we have shown that TA was able to target different signaling pathways through both the diminution and the activation of ERK1/2, AKT, and P38 kinases (Figure 7). Together, these events contribute to strongly delaying the tumor growth in mice, as demonstrated by the action of TA in the mice that were grafted with CT26 CRC cells.

## 5. Conclusions

Based on functional assays using both human and murine cancer cells, in this study we have explored the anti-metastatic impact of TA on CRC. Our results demonstrate that TA decreases cell motility/invasiveness by reducing the expression of the mesenchymal biomarkers β-catenin and E-cadherin, and by increasing that of the epithelial markers Slug, Snail, ZEB1, and N-cadherin. These molecular events were associated in vivo with a tumor regression in mice that were supplemented with TA, without apparent signs of hepato- and nephrotoxicities. These findings pave the way for the recommendation of nutraceutical TA or tannin-containing foods as preventive agents and potential adjuvants and as a complementary approach to conventional treatments in order to improve treatment outcomes.

## Figures and Tables

**Table 1:** TA IC_50_ values were determined after 24 h, 48 h, and 72 h of treatment in CRC cell lines. Statistical significance was determined by one-way ANOVA, followed by Tukey’s test for multiple comparison. *** *p* <0.001, **** *p* <0.0001 vs. CT26 cells at 24 h; ^####^ *p* <0.0001 vs. SW480 cells at 24 h; ^$$$^ *p* <0.001, ^$$$$^ *p* <0.0001 vs. CT26 cells at 48 h; ^££££^ *p* <0.0001 vs. SW480 cells at 48 h; ^¥¥¥¥^ *p* < 0.0001 vs. CT26 cells at 72 h; and ^&&&&^ *p* < 0.0001 vs. SW480 cells at 72 h.

Time of Treatment (h)	Cell Line		
	CT26	SW480	SW620
**24**	34.48 ± 1.20	48.49 ± 1.68 ***	88.17 ± 2.77 ****^, ####^
**48**	11.16 ± 0.14	12.16 ± 0.11 ^$$$^	44.20 ± 0.20 ^$$$$, ££££^
**72**	7.61 ± 0.14	9.40 ± 0.08 ^¥¥¥¥^	25.75 ± 0.02 ^¥¥¥¥, &&&&^

## Data Availability

The authors declare that all data supporting the findings of this study are available within the article.
